# Relationships Among Childhood Bullying, Academic Satisfaction, and Mental Health Outcomes in Adults with Disabilities

**DOI:** 10.3390/diseases13060165

**Published:** 2025-05-23

**Authors:** Bryan R. Christ, Bani Malhotra, Ghizlane Moustaid, Olivia Chapman, Paul B. Perrin

**Affiliations:** 1 School of Data Science, University of Virginia, Charlottesville, VA 22903, USA; brc4cb@virginia.edu; 2 Department of Physical Medicine and Rehabilitation, Virginia Commonwealth University, Richmond, VA 23298, USA; bani.malhotra@vcuhealth.org; 3 Department of Psychology, University of Virginia, Charlottesville, VA 22904, USA; afh6ys@virginia.edu (G.M.); qav3hs@virginia.edu (O.C.)

**Keywords:** disability, childhood bullying, academic satisfaction, adult mental health

## Abstract

Purpose/Objective: Children with disabilities are at a greater risk of being bullied and experience mental health and academic problems that may persist in adulthood. This study examined the association of childhood bullying experiences with current mental health (anxiety and depression) among adults with disabilities, and whether academic satisfaction mediated the relationship between childhood bullying and adult mental health outcomes. Research Method/Design: A sample of 409 adult participants with disabilities who had had their disabilities while attending school, and currently, completed an online survey assessing bullying experiences (California Bullying Victimization Scale-Retrospective), academic satisfaction (Academic Satisfaction Scale), depression (Patient Health Questionnaire-9), and anxiety (Generalized Anxiety Disorder-7). Bivariate correlations and two mediation analyses were conducted to identify the direct and indirect effects of school bullying experiences on current mental health outcomes, via academic satisfaction. Results: Participants reported a moderate amount of childhood bullying and relatively high levels of depression and anxiety symptomology (with averages close to or exceeding clinical cutoffs of 10). Bivariate correlations among the four variables were all significantly moderately or strongly correlated. Bullying and academic satisfaction had direct associations with depression and anxiety. Academic satisfaction partially mediated the relationships between bullying and both mental health outcomes. Conclusions/Implications: Bullying prevention interventions and programs, especially geared toward preventing bullying in students with disabilities, are critical to stop the likely long-term impacts of bullying on mental health outcomes in disabled communities.

## 1. Introduction

### 1.1. Rates of Bullying

Bullying is an unfortunately common adverse experience of childhood and adolescence in the U.S., with approximately 34% of teenagers experiencing some form of bullying in 2023 [1]. The most prevalent forms of bullying occur in relational, verbal, and physical domains [2,3]. Childhood bullying often takes place within school settings. During the 2021–2022 school year, 28% of middle schools, 15% of high schools, and 10% of elementary schools in the U.S. reported incidents of bullying that occurred at least once a week [4]. Teenagers with disabilities are particularly vulnerable, facing a 32% higher likelihood of being bullied compared to their peers without disabilities [5]. Students with disabilities are overall 282% more likely to be bullied because of their disability [5], with increased severity [6] and visible disabilities [7], further heightening their risk of victimization. Given that over 86% of students with disabilities face exclusion and rejection at school [8], and nearly 50% are subjected to mockery and insults based on their appearance [8], there is an urgent need for in-depth research on the potentially long-term effects of childhood bullying on individuals with disabilities.

### 1.2. Academic Satisfaction and Bullying

Non-disabled bullied youth experience poor academic outcomes [9,10], weakened school connectedness [9,11], decreased sense of belonging [12,13], and low academic self-perception [11]. However, many bullied students may also be resilient, as those who were bullied in the past have shown academic improvement over time [11]. Disabled bullied youth have been shown to experience similar negative academic experiences and outcomes as their non-disabled peers [14], with many expressing academic dissatisfaction as a result of the mental distress caused by bullying [15]. In addition, many students with disabilities often also lack vital social skills [16,17] and a strong peer-support network [18] to defend and protect themselves from their aggressors, placing them at a further disadvantage in school. Unlike their non-disabled peers [19], bullied youth with disabilities often report never confronting their bullies or reporting incidents to school administration or family, despite being aware of the negative impact on their educational performance [15], thus perpetuating a cycle of continued victimization.

### 1.3. Bullying and Mental Health

Bullying experiences lead to negative mental health outcomes, including depression [20], non-suicidal self-injury [21], anxiety [22], and PTSD [22] for all affected youth. Childhood bullying has long-lasting effects on mental health, with bullied youth facing higher rates of depression, anxiety disorders, and suicidality in adulthood compared to their non-bullied peers [23]. Similarly, bullied children with disabilities are at a greater risk for mental health issues (e.g., anxiety and depression) in adolescence and adulthood compared to non-bullied children with disabilities and children without disabilities [24,25,26]. The negative impact of disability on mental health is largely mediated by experiences of bullying and victimization, highlighting the significant role of disability-based bullying in driving negative mental health outcomes [27].

### 1.4. Academic Satisfaction and Mental Health

Low academic satisfaction is strongly associated with a variety of poor mental health outcomes, including depression, anxiety, and stress [28], while high academic satisfaction, in the form of academic achievement [29] and supportive school relationships [30] is associated with improved well-being. Overall life satisfaction mediates the relationship between school relationships and mental health, potentially mitigating the impact of low academic satisfaction [30]. The impact of childhood school experiences extends into adulthood, with negative experiences in adolescence leading to depressive effects later in life [31]. Conversely, mental health also affects academic satisfaction, as students diagnosed with depression, anxiety, or an eating disorder are 1.86 times more likely to be academically dissatisfied, with the likelihood doubling when all three conditions are present [32]. Although individuals with disabilities are at risk of academic dissatisfaction [15] and poor adult mental health outcomes [33], the long-term relationship between these factors remains understudied.

### 1.5. Purpose of the Current Study

While prior research has explored childhood bullying, academic satisfaction, and mental health in students with disabilities, no studies have examined the relationships among all three factors and whether that relationship is associated with adult mental health outcomes many years later. Because bullying and low academic satisfaction are both related to decreased adult mental health outcomes and bullying can decrease academic satisfaction, it is possible that all three factors might work together in a self-perpetuating cycle to affect mental health outcomes, perhaps many years into adulthood. In this cycle, bullying could directly lead to both adverse mental health outcomes and indirectly negatively impact these outcomes by decreasing academic satisfaction. Decreased academic satisfaction could then further inhibit psychosocial functioning above and beyond experiences of bullying. As a result, the purpose of this study was to probe at this theory by investigating the potentially long-term association of childhood bullying experiences with current mental health in adults with disabilities and examine whether academic satisfaction mediates this relationship.

## 2. Method

### 2.1. Participants

This study collected data from 409 adults who identified as having a disability or chronic health condition while going to school in the U.S. as well as currently. Participants’ ages ranged between 19–86 years old, with an average age of 39 (SD = 12.5). Fifty-one percent of the participants identified as women, 37% as men, 8% as gender non-binary, 2% as transmen, 1% as transwomen, and 1% as other. Participants self-identified as White (73%), Black/African-American (11%), Latina/o/x or Hispanic (6%), Multiracial/Multiethnic (6%), Asian/Asian-American/Pacific Islander (3%), and American-Indian/Native-American/Alaska-Native (1%). Further sample demographics are shown in Table 1.

### 2.2. Procedure

The Institutional Review Board from the host university granted approval for this study. All data were collected in two parts through Prolific, an online and secure data collection platform similar to Amazon’s Mechanical Turk that handles participant recruitment, prescreening, and payment. In the first part, nine-hundred and seventy individuals completed a one-minute initial screening survey to determine eligibility, as existing Prolific screening criteria do not account for whether individuals had a disability or chronic health condition while attending school in the U.S. In part two, all initially eligible individuals (*n* = 652) were sent a standardized invitation via Prolific inviting them to take part in a 30-min survey with the aim of exploring their school-based experiences. The second survey was completed on a first-come, first-served basis and was closed after reaching 419 complete responses, which was the maximum number of complete responses we were able to collect given our study’s budget. Eligible participants for the second survey identified as (1) being 18 years or older and (2) having a disability or chronic health condition while going to school in the U.S. and currently. Prior to participating in the study, all interested individuals were provided with an information sheet detailing the purpose of the study and provided informed consent. The second survey involved administering online self-report surveys lasting approximately 30 min. Participants received $0.14 for the screening survey and $6 for the full survey, for a total compensation of $6.14. The full survey included quality control questions that asked participants about their age at both the beginning and end of the survey. Additionally, participants were also asked an open-ended question (“In one sentence or less, what did you think was the purpose of this survey?”) at the end of the survey. Seven participants were removed due to failing both quality control checks. Three additional participants were also removed due to inconsistent reporting of their disability or chronic health condition from what they reported on the screening survey. This resulted in a final sample of 409 participants with complete survey data.

### 2.3. Measures

***Experiences of Bullying.*** The California Bullying Victimization Scale—Retrospective was used to assess participants’ past experiences of childhood bullying during their time in school [34]. Participants were asked about the frequency of eight different forms of bullying, including teasing, rumors, purposeful ignoring, physical harm, threats, sexual harassment, stealing or damage to personal belongings, and cyberbullying on a 7-point Likert scale ranging from 0 (never) to 6 (several times a week). These 8 items include statements such as “Been teased or called names in a mean or hurtful way” and “Been hit, pushed, or physically hurt in a mean or hurtful way.” We created a sum score of bullying, whereby higher scores indicated more severe self-reported childhood bullying.

***Academic Satisfaction.*** Participants were asked about their overall academic satisfaction in several key domains, including coursework, educational quality, and career preparation using the Academic Satisfaction Scale [35]. Items of the scale were slightly modified to incorporate both past and present tense, ensuring that the items were relevant for participants who are currently in school as well as those who were not. The 11-item scale includes statements such as “I was or am happy with the amount I learned or am learning in my classes” and “I was or am able to use my talents, skills, and competencies in my courses,” with responses measured on a 5-point Likert scale ranging from 1 (strongly disagree) to 5 (strongly agree). Despite the minor change to this scale, the Cronbach’s alpha in the current sample was 0.91. The scale has one item that is reverse coded: “My courses were or are not really what I liked or like to be doing.” We created a sum score of academic satisfaction, whereby higher scores reflected greater academic satisfaction.

***Mental Health.*** We assessed participants’ depression and anxiety symptoms within the past two weeks using the 9-item Patient Health Questionnaire (PHQ-9) [36] and 7-item Generalized Anxiety Disorder-7 (GAD-7) [37]. Participants were asked the frequency of their depression (e.g., “Feeling down, depressed, or hopeless”) and anxiety symptoms (e.g., “Feeling nervous, anxious, or on edge”) on a 4-point Likert scale ranging from 0 (not at all) to 3 (nearly every day). We created a sum score for each measure, with higher scores indicating a higher severity of symptoms.

### 2.4. Data Analysis

Analyses were conducted using SPSS 29 with the PROCESS macro. Descriptive statistics and basic assumption analyses (i.e., linearity of relationships, absence of multicollinearity, skewness, and kurtosis) were first conducted. Bivariate correlations were conducted to assess for multicollinearity associations among the primary study variables. This was followed by two mediation analyses where both the direct and indirect effects of retrospective experiences of bullying during the school years on current depression and anxiety symptoms were tested, separately, via academic satisfaction. Results were considered significant if the coefficient 95% confident interval (CI) did not include zero.

## 3. Results

### 3.1. Descriptive Statistics and Assumption Tests

Table 1 shows demographic characteristics of participants and their average academic satisfaction, bullying, depression, and anxiety scores. Participants had moderate academic satisfaction on average, although a wide standard deviation suggests there was much variability in their academic experiences. Most participants reported at least a moderate amount of childhood bullying, though the wide standard deviation again suggests much variability in their individual experiences. Participants reported relatively high levels of depression and anxiety symptomology, as the average score for both measures was close to the clinically relevant cutoff for depression/anxiety symptomology of 10 or higher on the PHQ-9 and GAD-7. Table 2 displays bivariate correlations among the four variables used in the two mediation models; notably, all included variables were either significantly moderately or strongly correlated. Given that the correlation between bullying and academic satisfaction was moderate, there was no evidence of multicollinearity. All skewness and kurtosis values were below an absolute value of one, and scatterplots among all variables indicated linearity.

### 3.2. Mediation Models

#### 3.2.1. Depression

**Figure 1:**
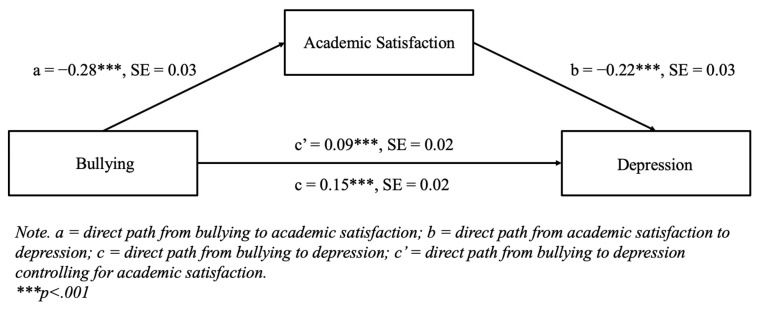
Mediation model for depression with standardized path loadings and standard errors using 5000 bootstrap samples.

Figure 1 displays the results for the mediation model for depression, where bullying was specified to have a direct effect on depression, as well as an indirect effect through academic satisfaction, using 5000 bootstrap samples. The direct paths from bullying to academic satisfaction (b = −0.28, *p* < 0.001) and from academic satisfaction to depression (b = −0.22, *p* < 0.001) were both statistically significant. The indirect effect of bullying on depression through academic satisfaction was statistically significant (b = 0.06, 95% CI [0.04, 0.09]), indicating a partial mediation because the direct path from bullying to depression was still statistically significant in the model (b = 0.09, *p* < 0.001).

#### 3.2.2. Anxiety

**Figure 2:**
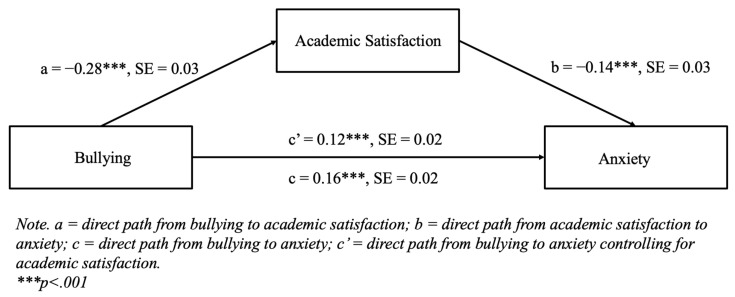
Mediation model for anxiety with standardized path loadings and standard errors using 5000 bootstrap samples.

Figure 2 displays the results for the mediation model for anxiety, where bullying was specified to have a direct effect on anxiety, as well as an indirect effect through academic satisfaction, using 5000 bootstrap samples. The direct paths from bullying to academic satisfaction (b = −0.28, *p* < 0.001) and from academic satisfaction to anxiety (b = −0.14, *p* < 0.001) were both statistically significant. The indirect effect of bullying on anxiety through academic satisfaction was statistically significant (b = 0.04, 95% CI [0.02, 0.06]), indicating a partial mediation because the direct path from bullying to anxiety was still statistically significant in the model (b = 0.12, *p* < 0.001).

## 4. Discussion

This study examined the relationships among childhood bullying, academic satisfaction, and current mental health in adults with disabilities. Consistent with prior research on the experience of childhood bullying experiences in individuals with disabilities, participants reported moderate amounts of childhood bullying and academic satisfaction though demonstrating high variability [5,6,15]. Particularly concerning in this sample were the high levels of mental health symptoms with depression and anxiety average scores being close to or above clinically significant (10 or higher). The bivariate correlations among the four variables were significant. Bullying showed a positive medium-sized correlation with depression and anxiety, and inverse correlation with academic satisfaction. These findings imply that adults who experienced bullying during their schooling may have exhibited lower academic satisfaction and enduring mental health effects into adulthood.

The finding that bullying is significantly related to negative outcomes for participants with disabilities are consistent with other studies, indicating that the harm from childhood bullying likely persists into adulthood. Aligning with the previous literature, higher rates of depressive and anxiety symptoms, and other psychiatric disorders among bullied youth, have been noted later in life [20,22,31,38], especially among those with disabilities [24,25,26,39]. For instance, [24] observed significant positive associations among bullying victimization, depression, and anxiety among autistic youth, non-autistic ADHD youth, and non-autistic non-ADHD youth with greater adverse outcomes noted among those who had a disability. The current study also found a wide range of academic satisfaction among the participants which was moderately associated with bullying experiences, in line with previous research on disabled victimized youth’s negative academic experiences and outcomes [9,14,15]. The variance noted in academic satisfaction could be due to differing experiences with bullying based on participants’ disability visibility or severity [6,7]; resources, supports, and accommodations available during schooling [40]; the regulatory frameworks governing educational inclusion when the participants attended schools [41]; or personal factors [42] and social factors [43] related to disability experience that may have implications for the students’ academic satisfaction.

The mediation path models tested in this study found bullying to have a direct association with depression and an indirect association through academic satisfaction leading to a partial mediation. Recent studies have demonstrated the relationship between bullying and depression [44], indicating its long-lasting effects on adult depression risk [38]. Furthermore, having a disability has been strongly linked to adverse mental health, with one study reporting bullying as mediating almost half (46%) of the total effect of disability [27]. The results of the current study are relevant because they indicate a clear relationship between childhood victimization and adult depression symptoms, and an indirect association through academic satisfaction. When a child with a disability is bullied, particularly when bullying is chronic, it may be persist over several years or across school transitions [45], and can adversely affect school and social functioning having longitudinal effects on psychosocial health, including depression, which subsequently impact concentration and educational attainment [23,46]. Likewise, in the current study, bullying had a direct association with anxiety and an indirect association through academic satisfaction among adults with disabilities. Consistent with prior studies, bullying has been shown to increase the risk of anxiety disorders [47] particularly in adulthood [38,48]. Experiencing mental distress caused by bullying can contribute to lower academic satisfaction and heightened anxiety among victimized disabled youth impacting individuals’ overall educational experience [15].

Notably, the partial mediational effects in the current study suggest that other variables should be examined when considering the intersections of childhood bullying with both adult anxiety and depression and how they interact with academic satisfaction. More research is needed to understand the mediating or moderating roles of personal and social factors in long-term mental health and the academic impact of bullying on individuals with disabilities. Personal factors, like self-esteem or self-concept [42,49]; social skills [17]; sense of belonging [14]; impact of disability microaggressions on identity development and internalized ableism [50]; loneliness and personality traits [51]; and gender, race, and participation in extracurricular activities [5,14], may be important variables in studying bullying among individuals with disabilities. Socio-ecological factors that could be considered for future research include peer social support as a predictor of decreased bullying [14]; poverty and neighborhood disorganization [44,52]; and accommodation, access, and support services [43]. This pattern of bullying’s diverse effects underscores the need to systematically examine the interplay of personal, educational, social, and community factors within the social/ecological framework of bullying and victimization [53].

## 5. Implications and Future Directions

The study provides important clinical, research, and policy implications, particularly regarding disability-related bullying and educational interventions. Future research in bullying prevention could consider examining unique experiences of different disability categories, using longitudinal designs to determine how disability type predicts bullying, and how interactions and associations vary over time. The findings underscore the importance of targeted bullying prevention programs and multifaceted interventions to reduce bullying among students with disabilities [54]. While the extant literature recognizes the risk factors associated with bullying at individual and school levels, further research is needed to identify factors associated with bullying and victimization for individuals with disabilities at community, institutional, and societal levels [14]. This study did not examine differences in the type of bullying experiences as relational or overt, or gendered experiences and their impact. Future research can consider exploring such intersectional associations. Despite the provision of antibullying laws and policies for students with disabilities, research on their effectiveness and implementation is missing. More research on institutional factors, such as the exposure and culture of school violence, vocational counseling for future career paths, and family involvement in special education, will provide insight on their roles in reducing bullying and its impact on academic satisfaction and mental health. Exploring protective factors, such as peer and teacher support, faculty awareness of accommodations, perspectives of disability service providers, and positive identity development, will shed light on ways to prevent and mitigate bullying [43,55].

The implications of research on bullying prevention interventions and institutional support are notable. By helping students, staff, and faculty recognize stigma and enhance social and communication skills, institutions and practitioners can proactively prevent negative peer interactions, ensuring emotional and academic support, and an overall inclusive educational environment. For instance, certain state mandates require public schools to teach students about the contributions of individuals with disabilities [56], and there have been efforts in fostering professional development aimed at the strength-based understandings of neurodiversity and antibullying resources for school staff, teachers, and administrators [57,58].

## 6. Limitations

There are several limitations to this study, including the retrospective reporting of bullying and educational satisfaction. Although this study highlights the impact of childhood bullying on current mental health, the emotional valence of participants’ experience may vary given the time elapsed between their bullying experiences and current responses. This could lead to recall bias or memory distortions especially when older participants reported childhood bullying and academic satisfaction. Additionally, the current study did not collect information on when participants had attended school and included a diverse age range for participants; given that rates of bullying may vary over time, this could lead to temporal mismatches in participants’ experiences of bullying based on when they had attended school. To reduce this potential bias, future well-resourced multidecade longitudinal studies could examine the associations among childhood bullying, academic satisfaction, and adult mental health outcomes using concurrent—and, therefore, more accurate—reports of school-related experiences. Furthermore, this study did not gather information on when the bullying occurred. Future research could examine the particular role of elementary, primary, or high school bullying experiences and their long-term impact among those with disabilities. The study’s reliance on online recruitment could have led to sampling bias, potentially limiting participation for individuals with certain disabilities and underrepresenting specific disability types or experiences. Participants in the study identified as having varied disabilities with the majority as experiencing difficulties in concentrating, remembering, or making decisions. The use of a six-item measure [59] to assess disability type is a limitation. While the study included participants who identified as having diverse limitations, it did not collect data on specific disability or disorders, making it difficult to examine the unique experiences of specific disability groups. Future research could examine differences in, or specific experiences of bullying among individuals with visible/invisible, chronic health or illness-related, psychiatric or neurodevelopmental disabilities [7,24,25]. Moreover, the interaction of mental health problems and bullying could be bidirectional, with early mental health issues being a risk factor of bullying [45]. This study did not account for participants’ childhood mental health problems, which could be important when examining the pathways of bullying and its impact on mental health issues or academic satisfaction later in life. Further, as this study only included two school-based factors that influence mental health—academic satisfaction and bullying—in building a mediational model, future work could consider adding additional factors, such as social support and school connectedness, to increase the complexity of the model and perhaps better explore the impact of childhood academic experiences on long-term mental health outcomes. Finally, the models tested did not account for potential demographic covariates that plausibly could have statistically influenced or accounted for some of the effects tested. While it is not statistical best practice to control for demographic variables that are not theorized to moderate or mediate other associations in the model, future research could consider controlling for possible confounds that researchers have reason to believe might influence similar models.

## 7. Conclusions

To date, there has been limited research in exploring the associations among bullying, academic satisfaction, and mental health outcomes among individuals with disabilities. This study offers preliminary evidence that childhood bullying may be associated with depression and anxiety directly and indirectly through academic satisfaction in adulthood among individuals with disabilities. The mediations observed underscore the importance of further examining these interactions with other personal and social factors that influence mental health and the educational experiences of disabled individuals. This study highlights the need for further research on bullying prevention and mitigation interventions, initiatives, and policies to enhance mental health and success among persons with disabilities.

## Figures and Tables

**Table 1:** Sample demographic characteristics and descriptive statistics.

**Variable**	**Mean**	**SD**
Age	39	12.5
Disability Index (out of 70)	30.2	13.3
Academic Satisfaction Scale (out of 55)	35.4	10.1
Bullying (out of 56)	18.3	14.2
Depression (out of 27)	11.3	6.9
Anxiety (out of 21)	9.5	6.0
	**Frequency (*n*)**	**Percentage (%)**
Category of Disability		
Deaf or Difficulty Hearing	47	11.5
Blind or Serious Difficulty Seeing	42	10.3
Difficulty Concentrating, Remembering, or Making Decisions	279	68.2
Difficulty Walking or Climbing Stairs	117	28.6
Difficulty Dressing or Bathing	57	13.9
Difficulty Doing Errands Alone	232	56.7
Gender		
Man	153	37.4
Woman	208	50.9
Transman	7	1.7
Transwoman	4	1.0
Gender non-binary/non-conforming	34	8.3
Other	3	0.7
Race/Ethnicity		
American-Indian/Native-American/Alaska-Native	4	1.0
Asian/Asian-American/Pacific Islander	13	3.2
Black/African-American	44	10.8
Latina/o/x or Hispanic	23	5.6
Multiracial/Multiethnic	24	5.9
White/European-American	300	73.3
Other	1	0.2
Educational Attainment		
Grade school (elementary or middle)	5	1.2
High school/GED	49	12.0
Some college (no degree)	111	27.1
2-year/technical degree	54	13.2
4-year college degree	151	36.9
Master’s degree	35	8.6
Doctorate degree	4	1.0
Urbanicity		
Urban	131	32
Suburban	187	45.7
Rural	91	22.2

**Table 2:** Correlation matrix among primary study variables.

Variable	1	2	3
1. Depression			
2. Anxiety	0.76 *		
3. Bullying	0.30 *	0.37 *	
4. Academic Satisfaction Scale	−0.39 *	−0.35 *	−0.40 *

Note. A * denotes a significant correlation at the *p* < 0.05 level.

## Data Availability

Due to privacy issues, data are available from the corresponding author upon request.
